# Facile, Efficient, and Cheap Electrode based on SnO_2_/Activated Carbon Waste for Supercapacitor and Capacitive Deionization Applications

**DOI:** 10.1021/acsomega.2c01458

**Published:** 2022-06-02

**Authors:** Ahmed S. Abou- Elyazed, Sameh Hassan, Asmaa G. Ashry, Mohammad Hegazy

**Affiliations:** † Chemistry Department, Faculty of Science, Menoufia University, Shebin El-Koom 32511, Egypt; ‡ MIIT Key Laboratory of Critical Materials Technology for New Energy Conversion and Storage, School of Chemistry and Chemical Engineering, Harbin Institute of Technology, Harbin 150001, China; § Physics Department, Faculty of Science, Menoufia University, Shebin El-Koom 32511, Egypt

## Abstract

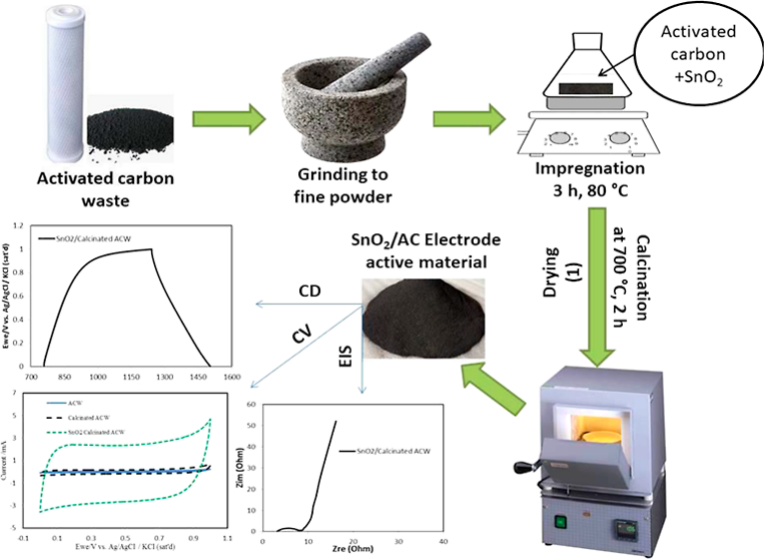

Activated carbon granules present in our household filters used in water purification are significant waste. Activated carbon waste (ACW) was ground to a fine powder, then impregnation of SnO_2_ on ACW was performed under mild conditions followed by calcination of SnO_2_-ACW at 700 °C for 2 h, producing a SnO_2_-ACW hybrid composite. This hybrid composite material was used in the preparation of electrodes for supercapacitor and capacitive deionization applications. The electrochemical performance of the electrodes was investigated by using cyclic voltammetry, galvanostatic charge–discharge, and electrochemical impedance spectroscopy. Calcination and addition of SnO_2_ contributed to an obtained electrode with a high specific capacitance of 30.46 F g^–1^ in a solution of 1 M Na_2_SO_4_ compared to the original ACW (0.122 F g^–1^) and calcined-ACW (1.42 F g^–1^) at an actual current of 1 mA. This electrode was also investigated for water desalination through the capacitive deionization technique and exhibited an electrosorption capacity of 6.44 mg/g compared to the commercial AC (8.9 mg/g) so it is a highly promising and economic electrode.

## Introduction

People use energy in everything, and this energy comes in several forms, some of which come in the form of heat and others in the form of radiation. Despite the different and multiple forms of energy, all of them can be classified under two types; either renewable energy or non-renewable energy; and the whole world is turning to the production of renewable energy of all kinds as an alternative to non-renewable energy, which affects one way or another on the climate and the environment.^1,2^ Therefore, renewable energies have become a pivotal part to reduce this risk, and examples of renewable energies such as sunlight, wind, rain, tides, and waves.^3^ Therefore, devices must be provided to store this energy until it is consumed when necessary. The most important examples of energy storage devices are supercapacitors.^4,5^

Supercapacitors are charge storage devices that received a lot of attention due to their high power density, excellent reversibility, and very long cycle life. Also, they deliver higher energy density than conventional capacitors and higher power density than batteries.^6,7^ In supercapacitors, active materials are the most essential factor in leading the electrochemical performance. In General, the electrode materials of supercapacitors are of three types, conducting polymers,^8,9^ carbon materials,^10^ and transition metal oxides.^11^ Each of them has its own merits and limitations.

Recently, the researchers have made more efforts to enhance the merits of the above-mentioned electrode materials and break the limitations by fabricating composites of metal oxides and carbon materials or metal oxides and conducting polymers. Among these efforts, Thangappan and co-workers investigated Mn–MoO_4_/graphene nanocomposite displays the specific capacitance (SC) (302.08 F g^–1^) at 0.1 A g^–1^ higher than that of graphene oxide (GO) (121.39 F g^–1^) and Mn–MoO_4_ (201.81 F g^–1^) in an aqueous electrolyte of Na_2_SO_4_.^12^ Liu and co-workers demonstrated a flexible and light-weight supercapacitor based on bacterial nanocellulose incorporated with tin oxide (SnO_2_) nanoparticles, GO, and poly(3,4-ethylenedioxyiophene)-poly(styrene sulfonate) (PEDOT: PSS) with a SC of 445 F g^–1^ at 2 A g^–1^.^13^

Generally, for double-layer capacitors, carbon-based materials are widely used as electrode materials. However, they exhibit only low SC and lower energy density than redox-based electrode materials. Metal oxides such as RuO_2_, NiO, SnO_2,_ and MnO_2_ have been studied by researchers with various structures of morphologies and the desired porosity structures as electrode materials for supercapacitors.^14,15^ Among the different metal oxides, SnO_2_ has exhibited a high value of SC and high cyclic stability, due to the highest theoretical capacity (∼782 mA h g^–1^), high abundance, and low cost.^16^

Additionally, other metal oxides like Ni, Mn, Sn, and Co materials have a high value in supercapacitor devices. However, the semiconducting property of these oxides is eradicating faster charge transfer at high current density and this attributes to reducing the rate capability.^17^ To solve this problem, many researchers investigated hybrid structures with high conductive carbon-based materials. Hence, binary or ternary composites have been widely investigated to obtain materials with the combined nature of properties in the balanced merit of the three kinds of materials. In this aspect, the activated carbon (AC) waste from household filter-based tin (VI) oxide composite is used as electrodes in both applications of supercapacitor and capacitive deionization (CDI).

In this study, a new two-step strategy has been followed for the synthesis of the SnO_2_-ACW composite. The first step involves the impregnation of SnO_2_ on AC waste under mild conditions, followed by the calcination of the composite at 700 °C for 2 h. The SC of the prepared composite is improved with excellent cyclic stability compared with the original AC waste. Additionally, it gives good results close to the commercial AC in the CDI process.

## Experimental Section

### Materials

Commercial AC, activated carbon waste (ACW), Tin (IV) oxide (SnO_2_, 99%), sulfuric acid (H_2_SO_4_, 98%), 304-stainless steel, *N*-dimethylpyrrolidone, polyvinylidene fluoride (PVDF), sodium sulfate anhydrous (Na_2_SO_4_, 99%) and distilled water. All chemicals are used directly without any further purification.

### Preparation of SnO_2_/ACW as an Active Material

**Figure 1:**
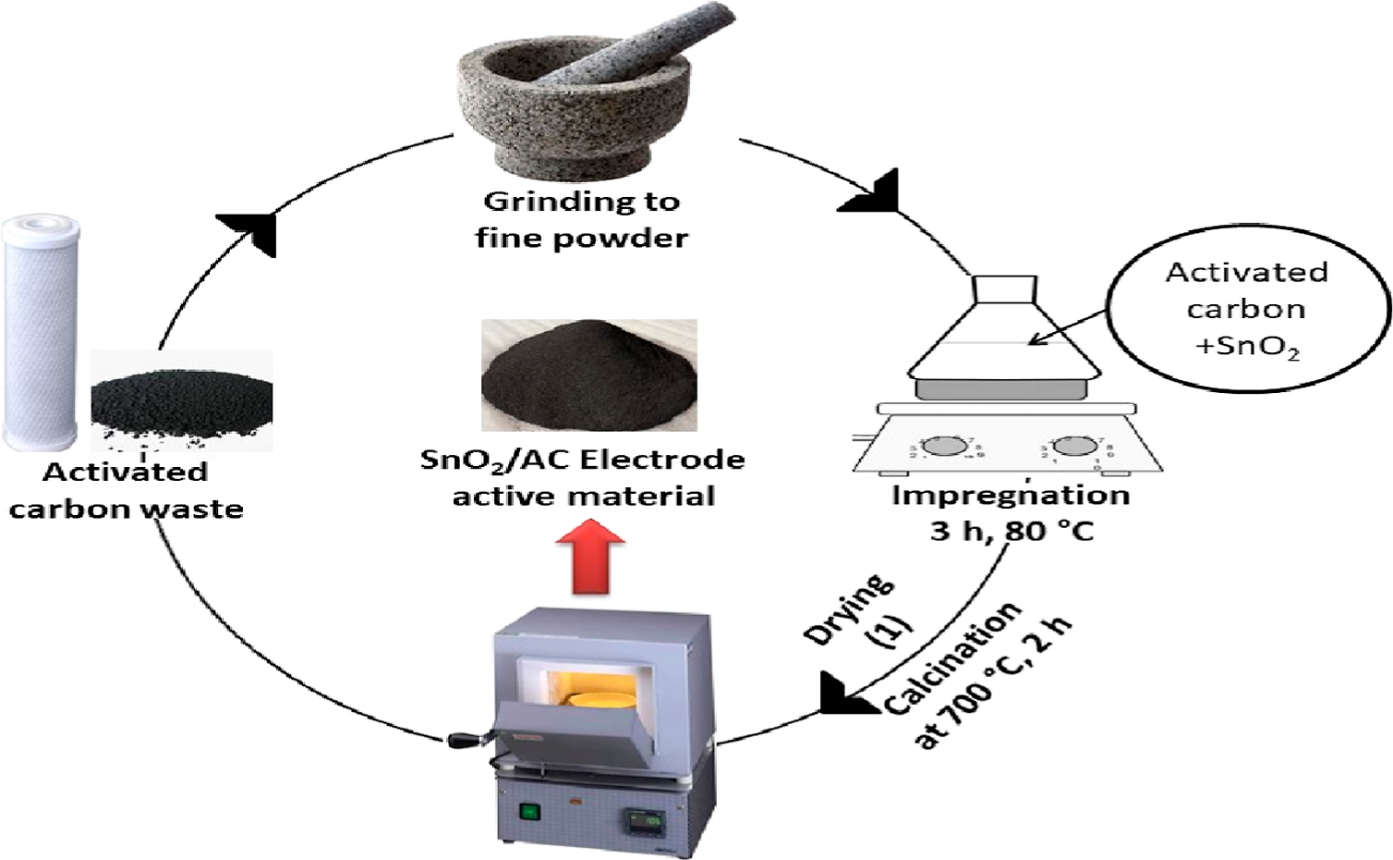
Active material preparation (SnO_2_/ACW) for energy storage and CDI applications.

The ACW from the house water filter was ground and prepared in three cases: ACW only, ACW-calcined at 700 °C for 2 h, and SnO_2_/ACW-calcined after the impregnation; where the SnO_2_ was impregnated on ACW in 50 mL glass vail for 3 h at 80 °C and then separated as black powder by centrifugation and drying in an oven for 2 h at 100 °C and then the powder was calcined for 2 h at 700 °C as shown in Figure 1.

### Electrode Preparation

The 304-stainless steel current collector was first etched using concentrated sulfuric acid 98% (3 mL) for 30 min, and then rinsed thoroughly with distilled water and then dried. The ACW electrode was prepared as follows; ACW, ACW-calcined, and SnO_2_/ACW-calcined (18 mg) as an active material, graphite (4 mg) as a conductive material, and poly-vinylidene-fluoride (PVDF, 2 mg) as a binder were mixed in a mass ratio of 75:17:8 with 0.4 mL of *N*-methyl-2-pyrrolidone as a solvent, and the mixture was sonicated in an ultrasonic bath at room temperature for half an hour to form a homogenous slurry. Further, the etched stainless steel was covered homogeneously from the above slurry by using a micro-pipette (10 μm) and then dried at 80 °C for 2 h to remove any remaining solvent. The mass loading of the prepared electrode for energy storage was measured utilizing a four-digit microbalance to be 9.4 mg (including graphite and PVDF mass) using an exposed area of 4 cm^2^. For CDI characterization, the CDI cell was investigated using the above-mentioned active materials as an electrode in a circular form with a diameter of 5.3 cm and a total active mass of 191 mg.

### Capacitive Behavior Test

The electrochemical behavior of the prepared electrodes was registered using Sp-150 potentiostat/galvanostat electrochemical workstation through cyclic voltammetry (CV), galvanostatic charge–discharge (GCD), and electrochemical impedance spectroscopy (EIS) techniques with a three-electrode system comprising a working electrode, a reference electrode, and a counter electrode. The prepared ACW electrode, Pt. wire, and Ag/AgCl (KCl saturated) electrode were used as a working electrode, a counter electrode, and a reference electrode, respectively. The CV experiments were performed at varying scan rates from 10 to 100 mV s^–1^ at the range of voltage (0–1 V). The GCD tests were recorded at actual currents from 1 to 10 mA within a voltage window from 0 to 1 V. EIS was performed at an alternating current amplitude of 10 mV superimposed on 10 mV DC voltage in the frequency range from 10 MHz to 100 kHz. The data was recorded through EC-Lab software V11.33 connected with the SP-150 potentiostat/galvanostat.

The SC, expressed in F/g, extracted from chrono-potentiometry discharge curves was calculated by using the charge–discharge current (*I*), voltage change with the time (Δ*V*/Δ*t*), and total mass (m) of electrode active materials using (eq 1) as follows
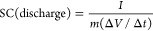
1

Cyclic voltammetry gravimetric capacitance (SC), expressed in F/g, was calculated by using the capacitive charge (*Q*), obtained from the integrated area of the CV curve divided by two, the active electrode total mass, and the width of the potential window (*V*) by using (eq 2).
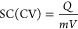
2

### Material Characterizations

Nitrogen sorption experiments were performed at −196 °C on a Quantachrome TouchWin version 1.2 Gas Sorption and Porosimetry system. The samples were regularly arranged for examination after degassing at 150 °C under a vacuum for 2 h until the final pressure reaches 1 × 10^–3^ Torr. X-ray diffraction (XRD) patterns were monitored on a Rigaku D/Max-2550 diffractometer furnished with a SolX detector-Cu K radiation with λ = 1.542 Å. Data were enrolled by step scanning at 2θ = 0.02° per second from 5 to 50°. Scanning electron microscopy (SEM) images were recorded on SEM quanta FEG 250 FEI and elemental determination from energy dispersive X-ray (EDX) was examined on (JCM-6000PLUS) fitted with an acceleration voltage of 15 kV.

## Results and Discussion

### Electrode Characterization

**Figure 2:**
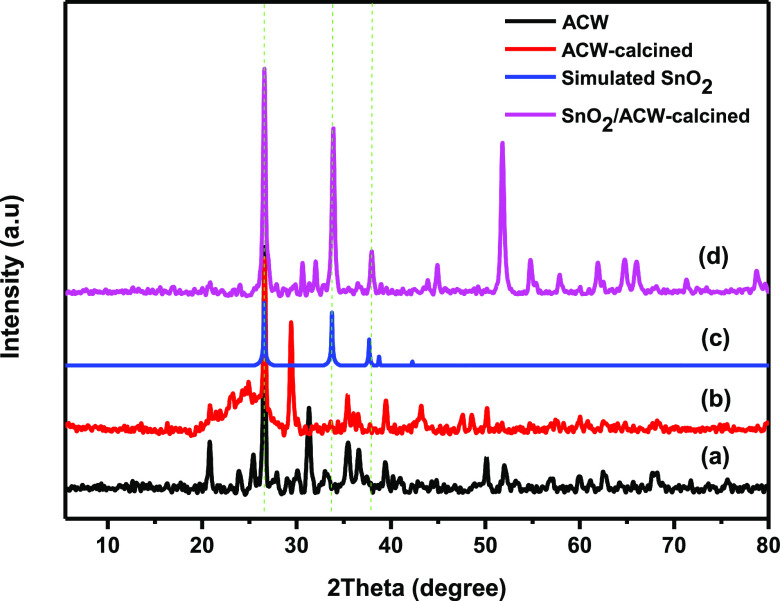
XRD patterns of (a) ACW, (b) ACW-calcined, (c) simulated SnO_2,_ and (d) SnO_2_/ACW-calcined samples.

Figure 2 displays the XRD patterns of ACW, ACW-calcined, simulated SnO_2,_ and SnO_2_/ACW-calcined samples. For the ACW from the household filter (Figure 2a), more broad diffraction peaks emerged corresponding to 2θ = 20.8, 26.7, 31.7, 35.4, and 39.5° in the spectrum. The XRD analyses of the ACW revealed that it appears in the amorphous state with low crystallinity, and the additional peaks were attributed to the contamination with different unknown materials. The ACW-calcined sample (Figure 2b), exhibits the disappearance of some diffraction peaks due to the vaporization of some contaminated materials from ACW after calcination at 700 °C. Also, the diffraction peak at 2θ = 25° becomes a broad peak compared to ACW owing to the effect of calcination temperature on the particle size of ACW. For SnO_2_/ACW-calcined sample (Figure 2d), three distinguished diffraction peaks were observed at 2θ = 26.6, 34, and 38°, respectively. These peaks are fitted with the standard JCPDS card of SnO_2_ (simulated SnO_2_, Figure 2) to confirm the successful preparation of the SnO_2_/ACW composite.

**Figure 3:**
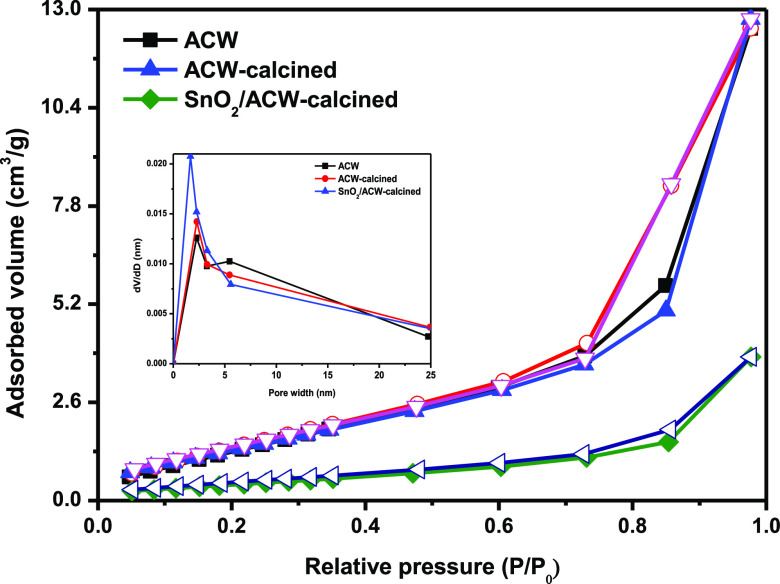
Adsorption–desorption isotherm and pore size distribution (inserted Figure) of various investigated samples.

N_2_ adsorption–desorption analysis was made to study the pore structure, pore-volume, and the specific surface area of the samples. N_2_ adsorption–desorption isotherms, pore-volume, and pore size distribution are shown in (Figure 3, Table 1). The isotherms can be classified as type IV, revealing the mesoporous structure of various prepared samples.^18,19^ From the adsorption branch of the isotherm, a slight increase in the specific surface area was observed by calcination at 700 °C (the specific surface for ACW, ACW-calcined, and SnO_2_/ACW-calcined samples were found to be 50.05, 56.89, and 56.45 m^2^ g^–1^, respectively). Table 1 indicates that the pore volume of ACW-calcined and SnO_2_/ACW-calcined samples exhibits wide pore volume compared to ACW sample, and the pore size distribution confirms the existence of mesopores and macropores^20^ as shown in Figure 3 (inserted Figure).

**Table 1:** Texture Properties of Different Samples

sample type	BET surface area (m^2^/g)	Langmuir surface area (m^2^/g)	pore volume (cm^3^/g)	pore size distribution (nm)
ACW	50.05	97.54	0.16	2.25–5.69
ACW-calcined	56.89	103.2	0.2	2.27–5.67
SnO_2_/ACW-calcined	56.45	101.5	0.19	1.67–1.69

**Figure 4:**
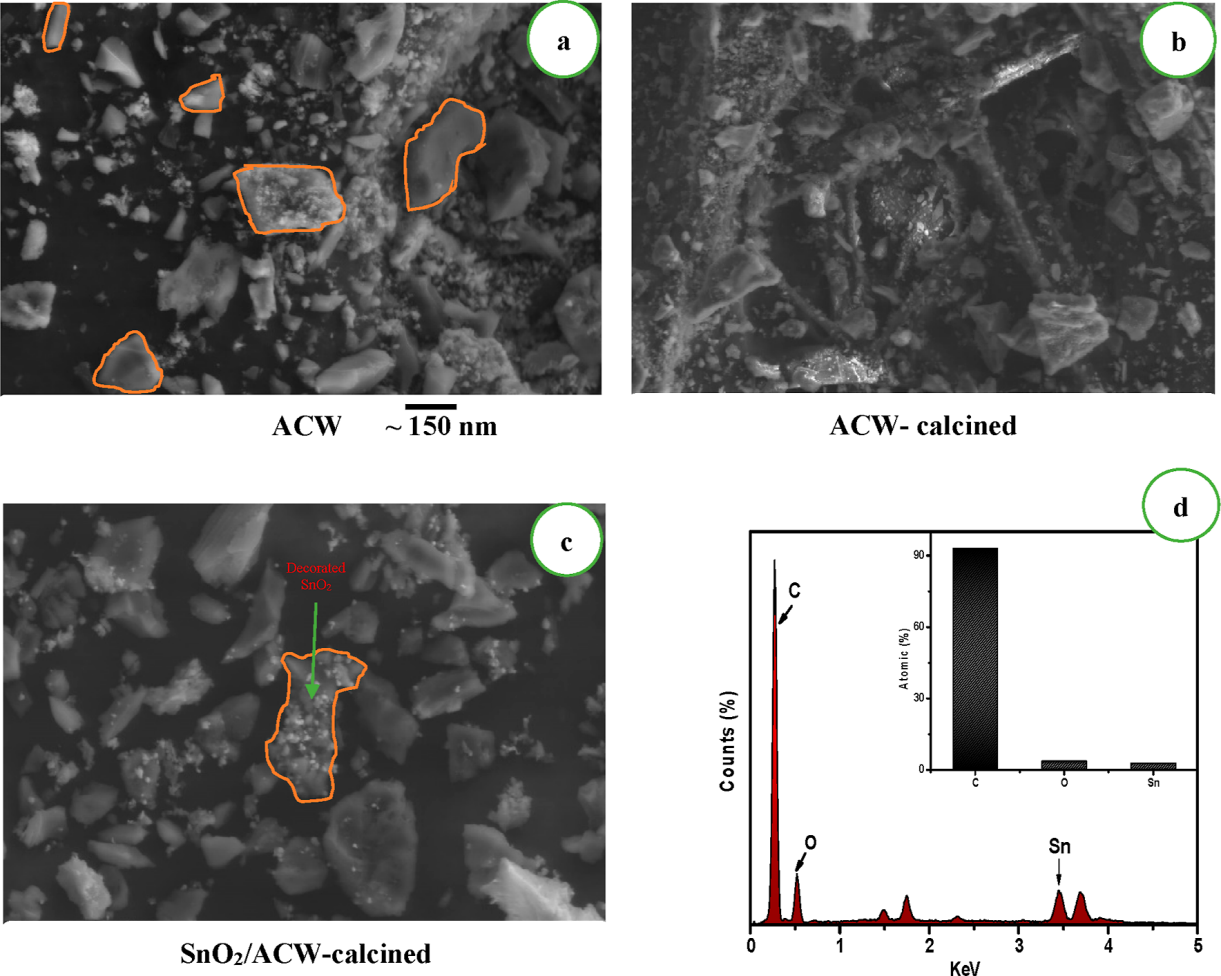
SEM images of (a) ACW, (b) ACW-calcined, (c) SnO_2_/ACW-calcined samples, and (d) EDX of SnO_2_/ACW-calcined sample. Electrochemical behavior of the electrodes.

The morphology of ACW, ACW-calcined, and SnO_2_/ACW-calcined samples was studied by SEM. Figure 4a shows the SEM image of ACW, it had irregular morphology with a size around 100–150 nm. After calcination at 700 °C for 2 h, the morphology changes to become a rod-like shape as shown in Figure 4b. In a close observation of the SnO_2_/ACW-calcined SEM image (Figure 4c), it was concluded that the particles of SnO_2_ were decorated on the ACW. Additionally, the EDX of the SnO_2_/ACW-calcined sample (Figure 4d) refers to the atomic content of Sn in the prepared sample after calcination.

The electrochemical performance of the electrodes was investigated in a three-electrode configuration in 1 M Na_2_SO_4_ electrolyte using CV, GCD, and EIS. Practically, the presence of SnO_2_ particles in the electrode of ACW resulted in a higher SC as compared to the original ACW as discussed below.

### Cyclic Voltammetry

**Figure 5:**
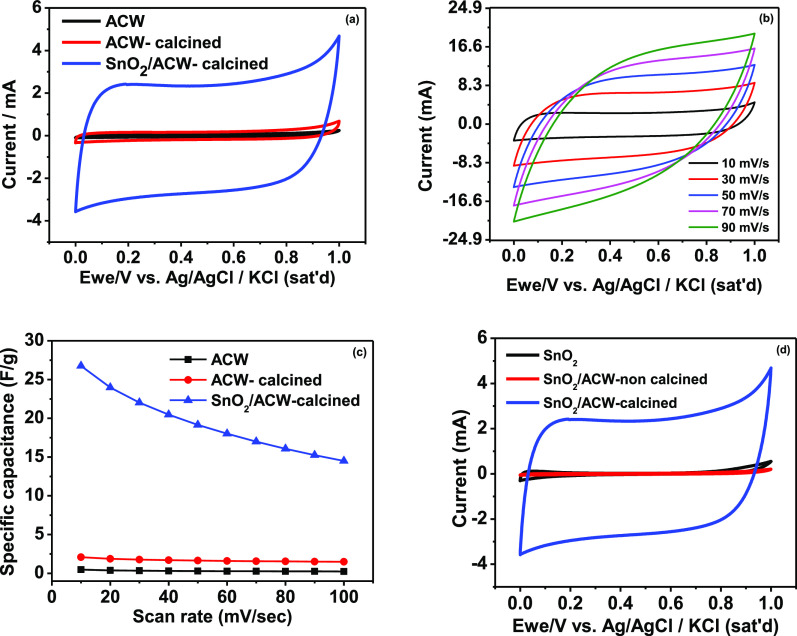
(a) CV curves at a scan rate of 10 mV s^–1^ for the prepared ACW electrode in all cases, (b) CV curves of SnO_2_/ACW-calcined electrode at different scan rates, (c) calculated SC for all investigated electrodes at different scan rates, and (d) CV curves for SnO_2_, SnO_2_/ACW-non calcined, and SnO_2_/ACW-calcined electrode at a scan rate of 10 mV s^–1^.

Figure 5a shows the common CV technique for the prepared ACW electrodes in the three cases in the potential window from 0 to 1 V at a scan rate of 10 mV/s in 1 M Na_2_SO_4_ electrolyte. The SC (as represented by the integrated area in the CV curve) of SnO_2_/ACW- calcined (26.75 F g^–1^) is significantly higher than that of ACW and ACW- calcined (0.48, 2.08 F g^–1^) respectively, due to highly reversible faradaic redox processes associated with SnO_2_ which was impregnated on the ACW support as confirmed by XRD and EDX. Also, the rectangular CV curve with an ideal double-layer behavior suggests a fast charge transfer rate even at high scan rates as shown in Figure 5b. The calculated SCs at different scan rates were given in Figure 5c in the common behavior of decreasing the SC with the scan rate. To ensure the capacitive behavior of the SnO_2_/ACW-calcined electrode, two electrodes were prepared as only SnO_2_ and SnO_2_/ACW without calcination and compared with the SnO_2_/ACW-calcined electrode. As seen, the SnO_2_/ACW-calcined electrode still has the larger integrated surface area giving higher capacitance which arises the effect of calcination on the SnO_2_/ACW composite material.

### Galvanostatic Charge–Discharge

**Figure 6:**
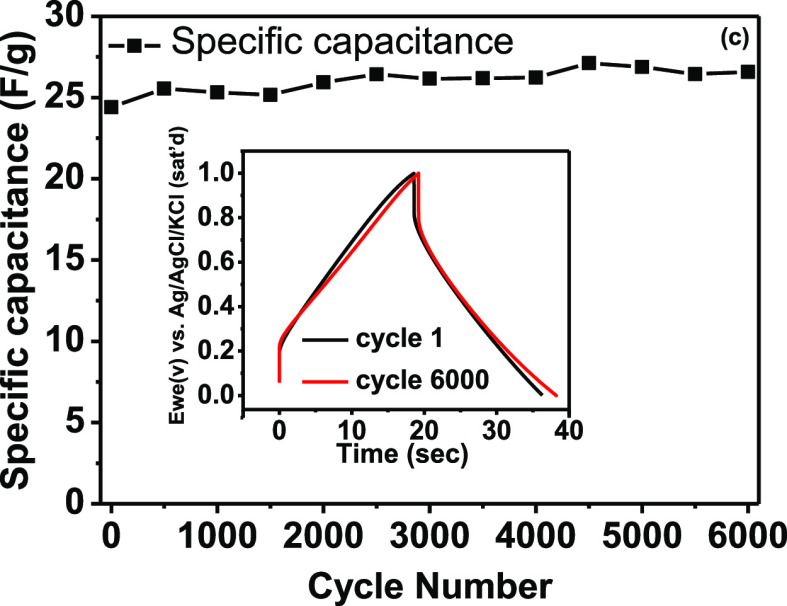
(a) CD curves at an actual current of 1 mA for the prepared ACW electrodes, (b) calculated SC for all investigated electrodes at different currents, and (c) the stability measurement at 10 mA for the SnO_2_/ACW-calcined electrode.

The capacitive performance of the prepared ACW electrodes in the three forms was also investigated through the GCD technique using 1 M Na_2_SO_4_ electrolyte at actual currents from 1–10 mA with a potential window between 0 and 1 V Figure 6a shows the GCD at 1 mA for the SnO_2_/ACW- calcined, ACW, and ACW- calcined. The voltage (ohmic) drop of SnO_2_/ACW-calcined electrode is very small compared with ACW and ACW- calcined indicating low DC internal resistance.^13,21^ Also, the slow discharge time of the calcined SnO_2_/ACW electrode expresses its high capacitance. Figure 6b indicates the calculated specific capacitance (*C*_sp_) variation with the actual charge–discharge current investigated in Na_2_SO_4_ electrolyte for the prepared electrodes in the three forms. Upon increasing the current, the SC decreases, which is a common behavior. The highest obtained SCs from the GCD curve were 0.122, 1.42, and 30.46 F g^–1^ at an actual current of 1 mA for ACW-based electrodes investigated in 1 M Na_2_SO_4_ electrolyte with three cases: ACW, calcined ACW, and calcined SnO_2_/ACW, respectively. The longevity and stability of the calcined SnO_2_/ACW electrode were evaluated using the GCD technique for 6000 cycles at a current of 10 mA (Figure 6c). The electrode retains 108.9% of its initial capacitance over 6000 charge–discharge cycles, indicating this composite electrode is a promising candidate for supercapacitor devices.

### Electrochemical Impedance Spectroscopy

**Figure 7:**
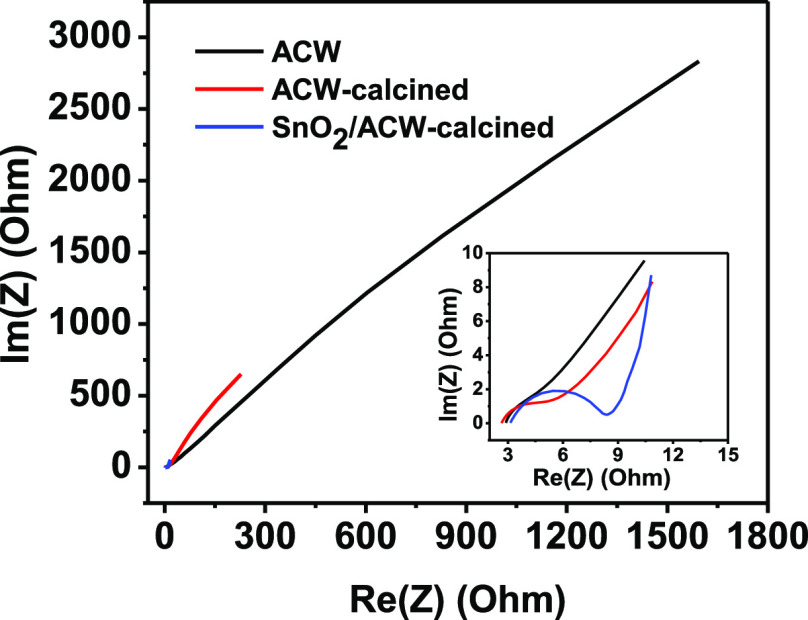
Nyquist plot of the ACW electrodes in three forms investigated in 1 M Na_2_SO_4_ electrolyte. The inset represents the zoom-in view of the Nyquist plot.

Figure 7 shows the obtained results of the EIS measurements, recorded in the frequency range of 10 mHz–100 kHz, applying 10 mV amplitude at room temperature using 1 M Na_2_SO_4_ electrolyte.

The observed plot shows different behaviors in the applied frequency range through the existence of a semi-circle in the high-frequency region and a straight line in the low-frequency region, indicating both the resistive and capacitive behaviors of the investigated electrode. The initial intercept of the semi-circle at the beginning with the real impedance axis at high frequency corresponds to the equivalent series resistance (ESR), which includes the ionic resistance of the electrolyte, interface resistance of the active material, the resistance of the current collector, and the contact resistance at electrode/electrolyte interface. The high-frequency intercept is followed by a semicircle due to the presence of resistance at the electrode–electrolyte interface and corresponds to the charge transfer resistance (*R*_ct_) that includes interfacial reaction kinetics. The electrode possibly blocks the electron exchange of the faradic process at the electrode/electrolyte interface. The value of *R*_ct_ can be derived from the diameter of the semicircle.^22^

The calculated ESR values were 2.89, 2.66, and 3.13 Ω for the developed electrodes in the three cases: ACW only, ACW-calcined, and SnO_2_/ACW-calcined, respectively. The result reflects a slight decrease in resistance after calcination of ACW due to larger pore volume and surface area of calcined particles resulting in fast ion diffusion, and then the resistance increased after the addition of SnO_2_. However, the values still reflect the high power density of the prepared electrodes. Also, the *R*_ct_ values followed the same trend with values of 5.51, 4.24, and 5.62 Ω. The pore structure variation in the smaller range of 1.67–1.69 nm with SnO_2_-ACW electrode (as indicated in Table 1) leads to a more accessible surface area which results in a higher SC.

### Capacitive Deionization Performance Evaluation

**Figure 8:**
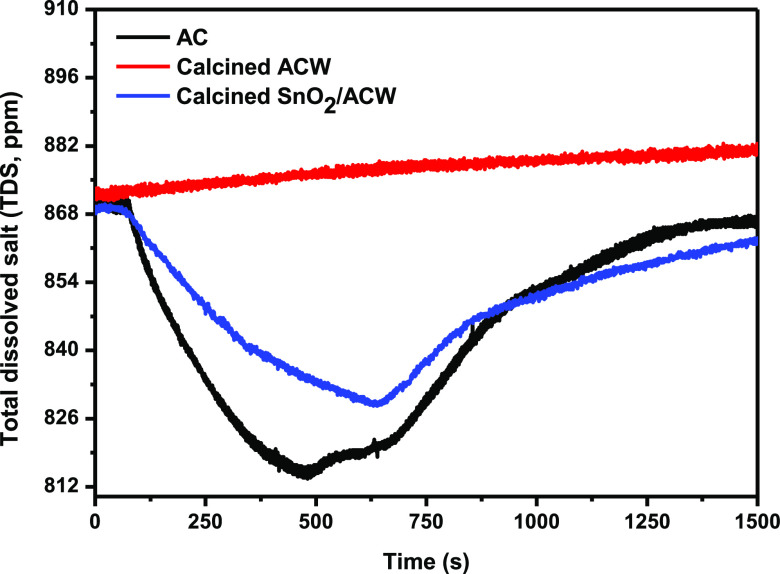
Variation of the TDS of NaCl solution with time during adsorption and desorption for the CDI cell electrodes.

To investigate the electrosorption capacity of the prepared ACW electrodes, a CDI cell was manufactured and the prepared electrodes were inserted between the cell plates without a separator between them. Figure 8 shows the change in the total dissolved solids (TDS) with time during adsorption and desorption for the CDI cell using the calcined ACW and SnO_2_/ACW- calcined as compared with the commercial activated carbon. As seen, except for calcined ACW, the TDS decreases rapidly with time during the voltage application where ions were quickly adsorbed on the electrode surface. For the calcined SnO_2_/ACW, NaCl electrolyte concentration decreased from the initial value of 870 ppm to a minimum of 828 ppm within 619 s at a desalination rate of 0.066 ppm/s. When all the accessible surface area was reached by the adsorbed ions, the electrosorption capacity of the CDI cell achieved the maximum value of 6.44 mg/g, and the salt removal efficiency reached 4.82% with the minimum concentration of NaCl solution. While in the case of commercial AC electrode cell, NaCl electrolyte concentration decreased from the initial value of 870 ppm to a minimum of 815 ppm within 447 s at a desalination rate of 0.127 ppm/s. The electrosorption capacity of the CDI cell achieved the maximum value of 8.93 mg/g, and the salt removal efficiency reached 6.52% with the minimum concentration of NaCl solution.

## Conclusion

In conclusion, a facile, abundant, and low-cost electrode was prepared from activated carbon waste and SnO_2_ by impregnation method followed by calcination at 700 °C for the supercapacitor device. The energy storage performance of the electrodes exhibited a significant improvement with the incorporation of SnO_2_ compared to the original ACW. The electrode exhibits excellent electrochemical performance with a specific capacitance of 30.46 F g^-1^ in an aqueous solution of 1 M Na_2_SO_4_ at the actual current of 1 mA. The stability of the calcined SnO_2_/ACW electrode was evaluated by obtaining GCD curves, and 108.9 % of its original capacitance was retained after 6000 cycles at the actual current of 10 mA. Also, this electrode with good capacitive behavior was investigated for capacitive deionization for water desalination giving a reasonable electrosorption capacity of 6.44 mg/g as compared with that of the commercial activated carbon (8.93 mg/g) with a promising adsorption and desorption behavior.

## Supporting Information Available

Supporting Information includes the experimental part for CDI, CD Figure of ACW/SnO_2_ before calcination, and SnO_2_ electrodes (PDF)
