# Supplementary material for: Facile, Efficient, and Cheap Electrode based on SnO2/Activated Carbon Waste for Supercapacitor and Capacitive Deionization Applications
Source: ACS Omega. 2022 Jun 2;7(23):19714–20. doi: 10.1021/acsomega.2c01458 (PMC9202029; doi:10.1021/acsomega.2c01458)
Supplement: Supplementary file 1 — ao2c01458_si_001.pdf [file ao2c01458_si_001.pdf]

Supporting Information

Facile, efficient, and cheap electrode based on SnO_2/activated carbon waste for supercapacitor and capacitive deionization applications

Ahmed S. Abou- Elyazed [a, b], Sameh Hassan [c, *], Asmaa G. Ashry [a], Mohammad Hegazy [a]

[a] Chemistry Department, Faculty of Science, Menoufia University, Shebin El-Koom, 32511, Egypt.

[b] MIIT Key Laboratory of Critical Materials Technology for New Energy Conversion and Storage, School of Chemistry and Chemical Engineering, Harbin Institute of Technology, Harbin, 150001, China.

[c] Physics Department, Faculty of Science, Menoufia University, Shebin El-Koom, 32511, Egypt

*Corresponding author**:** sameh.hassan@science.menofia.edu.eg, Tel: (+20)1001013552.

Experimental

Capacitive deionization (CDI) experiment

The CDI setup includes a reservoir, a peristaltic pump (LONGER BQ50-1J) to control the solution flow between the electrode pairs, a CDI unit cell, and a wireless conductivity meter (PS-3210) to detect the variations in the total dissolved solids (TDS) of the feed solution by SPARKvue software. Acrylic plates were used to assemble the CDI unit cell with lower and upper parts for inlet and outlet water feed, respectively. SP-150 Biologic potentiostat/galvanostat electrochemical workstation was used for the applied potential control and measuring the current response through EC-Lab software. The prepared active carbon waste electrodes were inserted into the CDI cell without separators or membranes, and their capacitive deionization performance was carried out in a continuous recycling system where the outlet water was returned to the feed reservoir (batch mode cell). The reservoir was a beaker filled with (30 ml) NaCl aqueous solution with a concentration of 870 ppm which was pumped through the CDI cell at a flow rate of 20 ml/min. A DC voltage of 2 V was applied for 600 s for adsorption, and this charging time was enough for electrode saturation. The regeneration of the electrodes was done by applying 0 V for another 600 s, where the adsorbed ions were returned to the solution.

Herein, the electrosorption capacity (Ce, mg/g) and the salt removal efficiency (η) in percentage for the CDI system were calculated by (eq.S1) and (eq.S2), respectively.

$$C_e = \frac{C_0 - C_f}{m} \times V \qquad \text{(eq.S1)}$$

$$\eta = \frac{C_0 - C_f}{C_0} \times 100 \qquad \text{(eq.S2)}$$

Where C_0 and C_f are the initial and final NaCl solution concentrations (mg/L or ppm), V is the volume of NaCl solution (L), and m is the total mass of active materials on the two electrodes (g). For each experiment, the test solution was fed to the CDI cell until the solution TDS becomes constant.

Galvanostatic charge-discharge

Figure S1 (a,b) shows the GCD at 1 mA for the SnO_2, SnO_2/ACW- noncalcined, and SnO_2/ACW-calcined electrodes, besides their calculated specific capacitance (Csp) variation with the actual

charge-discharge current, respectively. These results explain the role of the thermal treatment (calcination) in enhancing the electrochemical behavior of the SnO_2/ACW- calcined electrode.

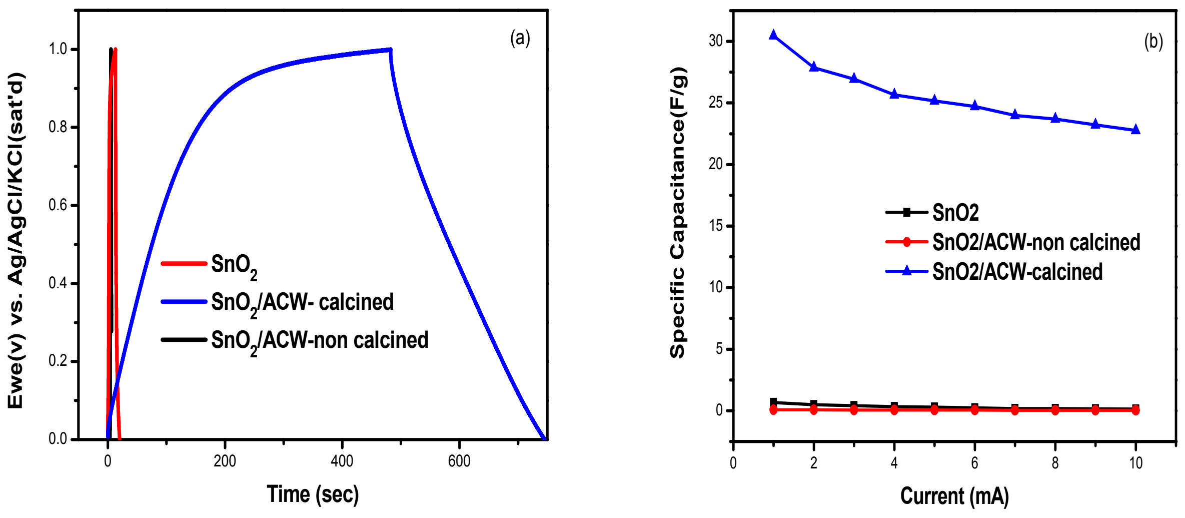

Figure S1. (a) CD curves at an actual current of 1 mA for the prepared various electrodes and (b) calculated specific capacitance for the prepared various electrodes at different currents.
